# Potential impacts of mercury released from thawing permafrost

**DOI:** 10.1038/s41467-020-18398-5

**Published:** 2020-09-16

**Authors:** Kevin Schaefer, Yasin Elshorbany, Elchin Jafarov, Paul F. Schuster, Robert G. Striegl, Kimberly P. Wickland, Elsie M. Sunderland

**Affiliations:** 1 National Snow and Ice Data Center, Cooperative Institute for Research in Environmental Sciences, University of Colorado Boulder, Boulder, CO USA; 2 School of Geoscience, College of Arts and Sciences, University of South Florida, St. Petersburg, FL USA; 3 Computational Earth Sciences, Los Alamos National Laboratory, Los Alamos, NM USA; 4 U.S. Geological Survey, Water Mission Area, Earth Systems Processes Division, Boulder, CO USA; 5 Harvard John A. Paulson School of Engineering and Applied Sciences and Harvard T.H. Chan School of Public Health, Harvard University, Cambridge, MA USA

**Keywords:** Biogeochemistry, Climate sciences, Ecology, Environmental sciences

## Abstract

Mercury (Hg) is a naturally occurring element that bonds with organic matter and, when converted to methylmercury, is a potent neurotoxicant. Here we estimate potential future releases of Hg from thawing permafrost for low and high greenhouse gas emissions scenarios using a mechanistic model. By 2200, the high emissions scenario shows annual permafrost Hg emissions to the atmosphere comparable to current global anthropogenic emissions. By 2100, simulated Hg concentrations in the Yukon River increase by 14% for the low emissions scenario, but double for the high emissions scenario. Fish Hg concentrations do not exceed United States Environmental Protection Agency guidelines for the low emissions scenario by 2300, but for the high emissions scenario, fish in the Yukon River exceed EPA guidelines by 2050. Our results indicate minimal impacts to Hg concentrations in water and fish for the low emissions scenario and high impacts for the high emissions scenario.

## Abstract (web summary)

Permafrost locks away the largest reservoir of mercury on the planet, but climate warming threatens to thaw these systems. Here the authors use models to show that unconstrained fossil fuel burning will dramatically increase the amount of mercury released into future ecosystems.

## Introduction

Naturally occurring and anthropogenic Hg deposits on land from the atmosphere and bonds to receptor sites in plant organic matter^1^. Microbial decay eventually consumes the organic matter^2^, releasing the Hg. Permafrost is soil at or below 0 °C for at least two consecutive years and the active layer is the surface layer of soil above permafrost that thaws in summer and refreezes in winter. Sedimentation in permafrost regions has buried vegetation over thousands of years, freezing organic matter at the bottom of the active layer into permafrost^3^. Once frozen, microbial decay effectively ceases, locking the accumulated Hg into the permafrost. Based on soil measurements, permafrost regions store an estimated 1656 ± 962 Gg Hg in the top three meters of soil, of which 793 ± 461 Gg Hg are frozen in permafrost^1^. Observations indicate accelerated permafrost thaw over the past 30–40 years^4,5^. Model projections estimate a 30–99% reduction in northern hemisphere permafrost extent by 2100^6,7^. When permafrost thaws, microbial decay of the stored organic matter will resume and release Hg, but how much, where, and when remain unclear.

Atmospheric deposition is the dominant source of Hg to the terrestrial biosphere^8,9^. Because Hg bonds to organic matter, the terrestrial carbon cycle modulates the terrestrial Hg cycle. Hg has three uptake pathways: (1) bonding to soil organic matter^8,10,11^, (2) stomatal leaf uptake^12,13^, and (3) root absorption^12,14^. Once absorbed by plants, translocation by phloem assimilates Hg into leaves and wood^12,13^. The deposition of dead leaves, roots, and stems transfers additional Hg to the soil^11,15^.

Hg has four release pathways: (1) evasion into the atmosphere after microbial decay^11^, (2) leaf stomata transpiration^14^, (3) fire^11^, and (4) leaching into groundwater followed by eventual export by rivers into the oceans^16^. Microbial decay frees Hg from organic matter, but plants and soil organic matter reabsorb^11^ most of this liberated Hg. Whether leaves represent an Hg source or sink depends on the concentration gradient between the stomata and the atmosphere^14^. Fire consumes soil organic matter, emitting carbon dioxide and Hg into the atmosphere^11^. Once leached into water, bound to Dissolved Organic Carbon (DOC) and Particulate Organic Carbon (POC), Hg can methylate, entering the food chain and accumulating in various species, particularly fish^8^.

**Fig. 1:**
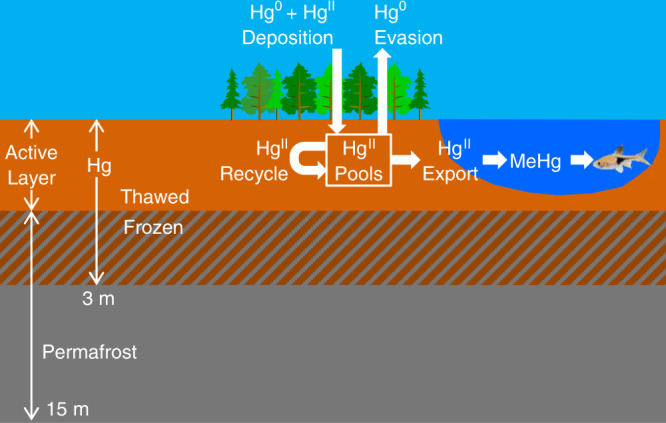
A schematic of our terrestrial mercury (Hg) model. The soil extends down to 15 meters with an active layer that thaws in summer and refreezes in winter. The organic carbon and Hg extend down to three meters. Hg deposits onto the surface from the atmosphere and bonds to plant and soil organic matter. As organic matter decays, elemental mercury (Hg^0^) is released into the atmosphere, some mercury cation (Hg^II^) is exported into rivers, and the remaining Hg^II^ is recycled back into the organic matter. We use empirical relationships to estimate methyl mercury (MeHg) concentrations in water from Hg^II^ export and total Hg concentrations in fish from MeHg concentrations.

The biological and physical processes that control the carbon cycle also control the Hg cycle^2,9,16^. We added Hg to the Simple Biosphere/Carnegie-Ames-Stanford Approach (SiBCASA) terrestrial biogeochemistry model^17^ (Fig. 1, Methods). The model accounts for all uptake pathways of Hg except leaf stomatal uptake. The model includes soil evasion and leaching Hg release pathways, but not stomatal transpiration and fire. We ran simulations from 1901 to 2299 using Representative Concentration Pathways 4.5 and 8.5 (hereafter RCP45 and RCP85). RCP45 represents a scenario close to the global target of 2 °C warming above pre-industrial levels while RCP85 represents a high emissions scenario of unconstrained burning of fossil fuels. We initialize the simulated Hg to a depth of three meters using observed values^1^ and ran the model for five thousand years to reach steady state where Hg losses balance gains. In 1901, SiBCASA simulates a total of 1370 ± 770 Gg Hg for all soils in permafrost regions, of which 863 ± 485 Gg Hg is frozen in permafrost, matching observed values within uncertainty^1^. SiBCASA simulates 507 ± 284 Gg Hg in the active layer, which agrees within uncertainty with observed estimates^18^ of 408 Gg Hg. SiBCASA accounts for the fact that microbial decay in frozen soil becomes limited to thin water films around soil particles^19^. Previous models did not account for this, resulting in high microbial decay and low soil Hg in permafrost regions^2^. Of the Hg released during microbial decay, we assume 16% evades into the atmosphere as gaseous elemental Hg^0^ ^2^, 1.5% is exported to rivers as the aqueous Hg^II^ cation^16,20,21^ and the remaining Hg is recycled back into plants and soil organic matter^2^. Based on observations, we assume 1% of Hg^II^ export methylates into the aqueous monomethyl mercury (MeHg)^16^. We estimate total Hg concentration in fish by multiplying the simulated MeHg by an empirical relationship of observed fish concentrations as a function of MeHg concentration in water (Supplementary Fig. 1).

We assume many processes remain constant that will likely change over time and space. For example, we assume constant atmospheric deposition of Hg^0^ to isolate the thawing permafrost signal, but changing anthropogenic and permafrost emissions will cause this to change over time. We assume constant Yukon River Basin (YRB) discharge, but warming temperatures and thawing permafrost will change the magnitude and timing of the seasonal water flow. We assume constant rates of DOC and POC leaching from soil, constant methylation rates, and steady accumulation of Hg in fish. However, large-scale permafrost thaw will change local hydrology, likely changing these rates^22,23^.

Here, we estimate potential future Hg releases from thawing permafrost for the circumpolar Arctic and impacts to Hg concentrations in water and fish in the YRB. We leverage a full-physics model of the terrestrial carbon cycle to simulate potential future changes in the terrestrial Hg cycle. We predict minimal Hg releases and small increases in Hg concentrations in water and fish for a low emissions scenario and large Hg releases and increases to Hg concentrations in water and fish for the high emissions scenario.

## Results and discussion

### Net Hg flux to the atmosphere

**Fig. 2:**
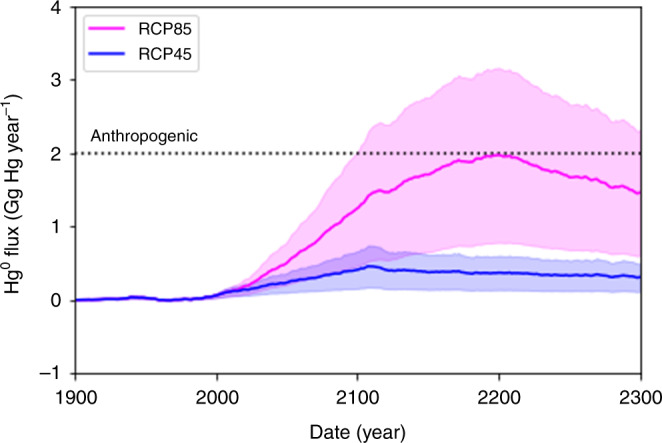
Annual net elemental mercury (Hg^0^) flux into the atmosphere. The net flux is Hg^0^ evasion into the atmosphere minus Hg^0^ deposition from the atmosphere, summed across all permafrost regions. The shaded areas represent uncertainty in the net Hg^0^ flux and the dashed line represents current global anthropogenic emissions.

Our simulations indicate thawing permafrost will result in net Hg^0^ fluxes to the atmosphere from permafrost regions comparable to the current total global anthropogenic emissions by 2200 under RCP85^24,25^ (Fig. 2). The net Hg^0^ flux is the Hg^0^ evasion into the atmosphere minus Hg^0^ deposition from the atmosphere. The net Hg^0^ fluxes hover around zero during the 1900s, indicating a rough balance between Hg^0^ deposition and evasion. The permafrost begins to thaw after 1980 (Supplementary Fig. 2) and by 2000 the decay of previously frozen organic matter increases, along with net Hg^0^ fluxes. By 2300, 29% of the permafrost in the top three meters of soil thaws for RCP45 and 84% of permafrost thaws for RCP85. For RCP45, the net Hg^0^ flux increases to 0.5 ± 0.3 Gg Hg year^−1^ and permafrost regions become a weak source of Hg^0^ to the atmosphere. For RCP85, the permafrost regions become a strong source of Hg^0^ to the atmosphere, peaking at 1.9 ± 1.1 Gg Hg year^−1^, comparable to current global anthropogenic emissions^8^ of ~2 Gg Hg year^−1^. Most of the net Hg^0^ fluxes result from the decay of old, currently frozen organic matter, so Fig. 2 indicates an injection of pre-industrial Hg into the modern Hg cycle. By 2300, we estimate permafrost regions will contribute a cumulative total of 101 ± 57 Gg Hg for RCP45 and 428 ± 244 Gg Hg for RCP85 to the global Hg cycle (Supplementary Fig. 3).

Our model does not include atmospheric transport, so we cannot predict where and when Hg^0^ emitted from permafrost regions will redeposit onto the surface. The total, pan-Arctic net Hg^0^ flux for RCP85 appears consistent in magnitude with global total anthropogenic emissions. However, unlike point sources typical of anthropogenic Hg, we expect to see the net Hg^0^ flux distributed across the vast Arctic landscape. Although thawed, the organic matter will decay slowly with a characteristic time scale of 75 years due to wet soils and periodic refreezing^26^. Once the permafrost thaws and the decay of organic matter resumes, Hg^0^ release from permafrost regions will likely persist for centuries.

The vegetation and soil organic matter act as a buffer to slow Hg^0^ evasion. In our model, 82.5% of the Hg released due to microbial decay recycles back into the organic matter. This recycling explains why we see a 30-year lag between the start of large-scale permafrost thaw in the 1990s and the increase in Hg^0^ evasion after 2020. Continual Hg deposition onto the surface also partially compensates for losses due to microbial decay. SiBCASA accounts for the northward migration of the tree line and enhanced photosynthesis due to warmer temperatures, longer growing seasons, and higher atmospheric carbon dioxide concentrations. This increased photosynthesis compensates for the loss of soil organic matter in thawed permafrost, resulting in a relatively constant ratio of Hg to carbon in plants (Supplementary Fig. 4).

### Spatial distribution of Hg losses

**Fig. 3:**
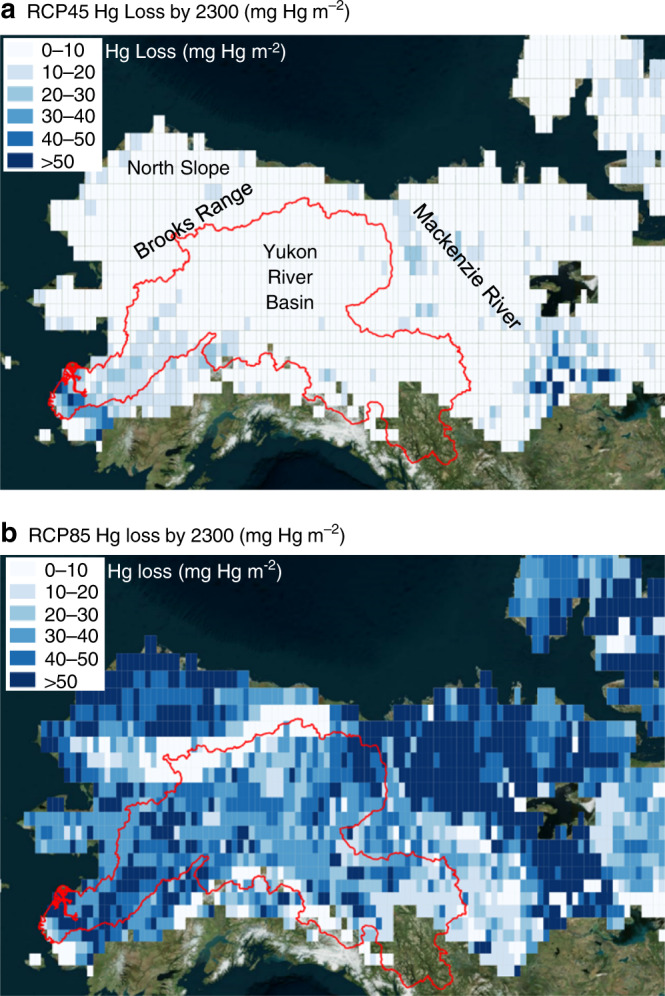
Cumulative mercury (Hg) loss per area by 2300. Total Hg loss is net elemental mercury (Hg^0^) flux to the atmosphere plus mercury cation (Hg^II^) export by rivers summed from 1901 to 2300. The red outline indicates the spatial extent of the Yukon River Basin.

Projected Hg losses occur in areas with the greatest thaw and the largest amount of frozen carbon (Fig. 3). For RCP45, permafrost thaw occurred along the entire southern margin of permafrost regions in the discontinuous permafrost zone. However, the greatest Hg losses occur only in thawed areas with large amounts of frozen carbon, specifically the lower YRB near the delta and the upper Mackenzie basin in Canada. This estimate does not account for Hg losses in areas susceptible to thermokarst or soil collapse during thaw, which our model does not include^27^. RCP85 showed extensive thaw everywhere with small Hg losses in areas with little frozen carbon, such as the Brooks Range, and the largest Hg losses in areas with the most frozen carbon, like the North Slope, Alaska. The other major rivers in North America and Eurasia showed the same pattern of Hg losses in areas of greatest thaw and the largest amount of frozen carbon (Supplementary Fig. 5).

### Export of Hg by the Yukon River

**Fig. 4:**
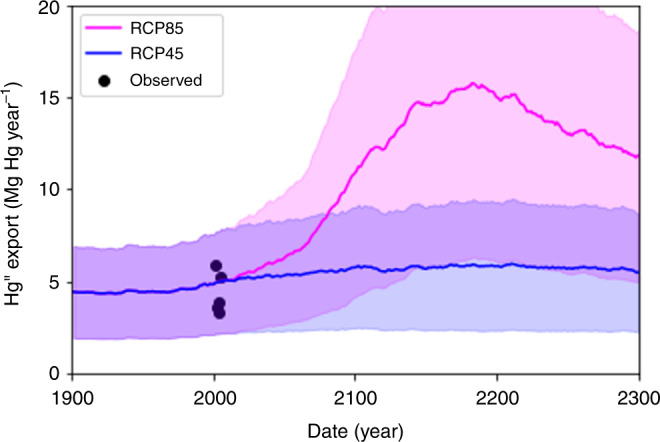
Mercury cation (Hg^II^) export for the Yukon River Basin (YRB). The annual riverine Hg^II^ export is the sum of Hg^II^ export across the YRB outlined in Fig. 3. The shaded areas represent uncertainty and the black dots indicate observed riverine export^16^.

The simulated export of aqueous Hg^II^ in the YRB shows small increases for RCP45 and large increases for RCP85 (Fig. 4, Supplementary Fig. 6). In 2003, the simulated Hg^II^ export for the YRB is 4.8 ± 2.7 Mg Hg year^−1^ while the observed export derived from in situ measurements is 4.4 ± 0.7 Mg Hg year^−1^, indicating our model matches observations within uncertainty^16^. RCP45 shows relatively small increases in Hg^II^ export, consistent with permafrost thaw primarily in the lower Yukon near the delta. In contrast, RCP85 shows permafrost thaw throughout the YRB resulting in a doubling of Hg^II^ export by 2100 and a tripling by 2200.

### Hg concentrations in the Yukon River

**Fig. 5:**
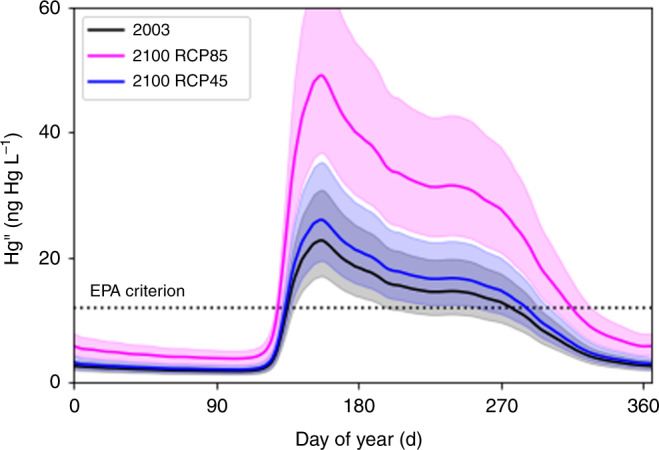
Mercury cation (Hg^II^) concentrations in the Yukon River. We calculated daily Hg^II^ concentrations at Pilot Station, Alaska on the Yukon River. The shaded areas represent uncertainties and the dashed line represents the former EPA ambient water quality criterion^28^ of 12 ng Hg L^−1^.

We compared simulated Hg concentrations in the Yukon River to the United States Environmental Protection Agency (EPA) former ambient water quality criterion^28^ of 12 ng Hg L^−1^ (Fig. 5). We see a minimum Hg^II^ concentration in winter when ice covers the river and a peak in spring when snowmelt increases discharge and Hg^II^ export. The Hg^II^ concentrations decrease by mid-summer to a relatively constant value and then further decrease in fall when the Yukon River begins to freeze over. In 2003, 85% of observed Hg^II^ concentrations in summer exceeded 12 ng Hg L^−1^ while we simulate nearly 100% exceedance. Nevertheless, the simulated Hg^II^ concentrations appear consistent with observed values^16^ (Supplementary Fig. 7).

Warming due to climate change will increase Hg concentrations in the Yukon River, particularly for RCP85 (Fig. 5). We simulate large-scale thawing throughout the YRB along with the appearance of taliks or layers of unfrozen ground where microbial decay and associated Hg^II^ export can persist throughout winter. For RCP45, we simulate limited thawing in the YRB. Hg^II^ concentrations increase by 14% in 2100 compared to 2003, a change well within the 30% uncertainty of simulated values. In contrast, the Hg^II^ concentrations for RCP85 increase by 116% in 2100 compared to 2003, more than double 2003 values. For RCP45, the number of days per year that exceed 12 ng Hg L^−1^ increase from 132 days to 152 days by 2100 (Supplementary Fig. 8). RCP85 exceeds 12 ng Hg L^−1^ for 188 days per year. For RCP85, Hg concentrations peak around 2160 (not shown), with 208 days exceeding 12 ng Hg L^−1^.

### Hg concentration in fish

**Fig. 6:**
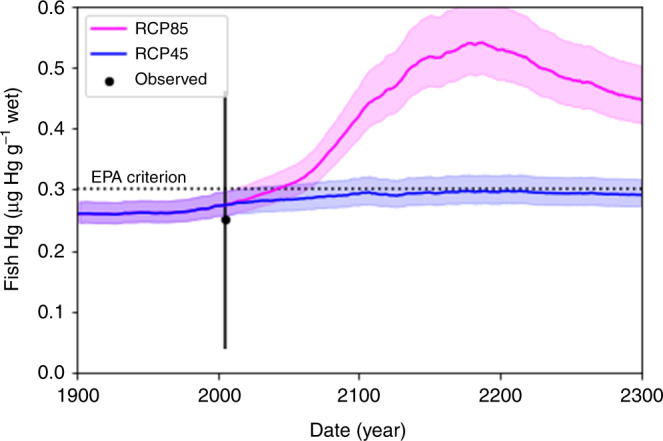
Mercury (Hg) concentration in fish in the Yukon River. The shaded areas represent uncertainty in the simulated values and the dashed line represents the EPA criterion. The dot and vertical error bar shows the median and range of observed concentrations in fish^29,30^.

Large-scale permafrost thaw may increase the concentration of Hg in fish (Fig. 6). We estimate the concentration of total Hg in fish (Hg_fish_) from simulated MeHg (Supplementary Figs. 1 and 9). The simulated Hg_fish_ falls well within the expected range of observed values^29,30^. Hg_fish_ varies among fish species and our estimates represent an average for all fish. For RCP45, the average Hg_fish_ only increases by 21% by 2100 due to limited thawing in the YRB. However, for RCP85, Hg_fish_ increases by 175% by 2100 and 222% by 2300. The EPA based its criterion of 0.3 g Hg g^−1^ wet weight on the reference dose for MeHg assuming average consumption rates^31,32^. For RCP45, Hg_fish_ does not exceed 0.3 g Hg g^−1^ wet weight, but for RCP85, Hg_fish_ exceeds 0.3 g Hg g^−1^ wet weight by 2150.

### Summary

Our results indicate large impacts to Hg concentrations in water and fish in the YRB for the high emissions scenario and minimal impacts of Hg contamination for the low emissions scenario. The Hg^0^ annual, pan-Arctic flux to the atmosphere by 2100 for RCP85 is nearly double that of RCP45 and consistent in magnitude to current anthropogenic fluxes. RCP85 has twice the Hg^II^ peak riverine concentration and annual Hg^II^ export for the YRB in 2100 compared to RCP45. By 2100, RCP85 exceeds EPA water quality criterion for the entire spring, summer, and fall, while RCP45 shows no significant increase in Hg concentration in water. Our results indicate a modest or small increase in Hg concentrations in water and fish in permafrost regions under RCP45. However, for RCP85, the scenario of unconstrained burning of fossil fuels, our results indicate substantial increases in Hg concentrations in water and fish due to the release of Hg from thawing permafrost.

## Methods

### Model overview

We estimated Hg releases from permafrost regions using projections of the Simple Biosphere/Carnegie-Ames-Stanford Approach (SiBCASA) model. SiBCASA is a full-physics land surface parameterization with fully coupled carbon, water, and energy cycles^17^. SiBCASA has a full soil model with prognostic soil temperature and moisture with 25 layers extending down to 15 meters. The permafrost dynamics and soil biogeochemistry models account for (1) the effects of soil organic matter on soil properties, (2) wind compaction of snow, (3) frozen soil biogeochemistry, (4) a dynamic organic layer, and (5) liquid water content in frozen soils^26,33–36^.

### Model pool structure

SiBCASA assumes vegetation growth, death, and microbial decay control Hg uptake and release from the terrestrial ecosystem^2^. We divide the carbon and Hg into 13 pools representing different vegetation and soil components, such as leaves or humus^17^. The flow of carbon from one pool to the next represents the life cycle of organic matter: photosynthesis produces starch, which plants use to grow leaves, roots, and wood. When the plants die, they produce litter and woody debris, which microbes consume to produce humus. Each time carbon and Hg transfers from one pool to another, some is lost as respiration of CO_2_ and evasion of Hg^0^ to the atmosphere. Each soil layer contains a complete set of carbon and Hg pools. SiBCASA accounts for the effects of soil temperature and moisture on microbial decay. In frozen soil and permafrost, SiBCASA limits microbial decay to thin water films surrounding soil particles^35^. Here, we report total Hg losses and gains summed over all pools and all layers within the soil column.

### Simulated Hg pathways

We assume total atmospheric Hg deposition consists of 71% Hg^0^ and 29% Hg^II^ based on observations^9^. SiBCASA has two uptake pathways, bonding to soil organic matter and root absorption. We assume all Hg gets oxidized to Hg^II^ during uptake and evenly distribute the Hg^II^ between the two pathways. SiBCASA has two release pathways: aqueous export of Hg^II^ by rivers and evasion of Hg^0^ to the atmosphere after microbial decay. We assume Hg^II^ export to rivers results primarily from leaching of Dissolved Organic Carbon (DOC) and Particulate Organic Carbon (POC). We assume a DOC leaching fraction of total respiration of 1.5 × 10^−3^ g C g C^−1^ based on laboratory measurements and a POC to DOC ratio of 9:1 to get a total aqueous Hg^II^ export fraction of 1.5%^16,20,21^ or 1.5 × 10^−2^ g Hg^II^ g Hg^−1^. After microbial decay, we assumed an atmospheric Hg^0^ evasion fraction of 16%^2^ or 0.16 g Hg^0^ g Hg^−1^. The remaining 82.5% of released Hg recycles back into the organic matter. We neglect Hg emissions due to fire and other potential Hg sources, such as glacial melt and leaching from bedrock.

We estimated Hg^II^ export for the Yukon River Basin (YRB) by summing Hg^II^ export for all pixels in the YRB and compared the result with observed values of Hg^II^ export^16^. We estimated an annual average Hg concentration in the Yukon River by dividing the annual export by the observed average discharge rate at Pilot Station, Alaska. We assumed daily Hg concentration is proportional to the discharge rate^16^ and used the median seasonal cycle in observed discharge rates measured at the Pilot Station gaging station^37^. We estimated methyl mercury (MeHg) concentrations using the observed ratio of 1%^16^ (Supplementary Fig. 9). We estimated fish Hg concentrations (Hg_fish_) by multiplying simulated MeHg by a regression of observed Hg_fish_ to observed MeHg (Supplementary Fig. 1).

### Uncertainty

We calculated uncertainty in all estimated parameters using Gaussian error propagation. We assumed independent errors and combined them in quadrature. The largest single source of uncertainty comes from the map of soil Hg used to initialize our model^1^. The next largest source of uncertainty comes from variability in predicted climate, represented as the standard deviation in simulated Hg fluxes for the five Coupled Model Intercomparison Project (CMIP5) simulations. The spread between models increased over time such that climate uncertainty varied from zero in the modern era to a peak of 37% after 2200. We calculated uncertainties for all regressions as the root mean square error (RMSE) of the residuals between the regression model and the original data. The RMSE varied between 5 and 15% for the various regression coefficients and model parameters, but these did not strongly contribute to overall uncertainty. The largest sources of uncertainty dominate overall uncertainty because we combine them in quadrature using Gaussian error propagation. The overall uncertainty varied from 30 to 60% and tended to increase over time, reflecting the increased uncertainty in climate. The largest, single source of uncertainty in these projections is the amount of soil Hg in permafrost regions.

### Model simulations

We ran simulations from 1901 to 2299 with a spatial resolution of 0.5 × 0.5 degrees latitude and longitude for the permafrost domain using output from five models in the fifth CMIP5 for Representative Concentration Pathways (RCP) 4.5 and 8.5 (RCP45 and RCP85 in the main text). We initialized the Hg pools down to three meters depth with a map of permafrost Hg based on observations^1^ and spun up the model for 5000 years to steady state initial conditions in 1901 with randomly selected weather years between 1901 and 1910. Steady state initial conditions in 1901 assumes Hg^0^ and Hg^II^ deposition from the atmosphere balances Hg^0^ evasion and Hg^II^ export. The resulting Hg atmospheric deposition rate of 0.75 Gg Hg year^−1^ or 0.9 ng Hg m^−2^ h^−1^ appears consistent with observed values of 1.4 ± 1.0 ng Hg m^−2^ h^−1^ on the North Slope of Alaska^9^. The simulations start at steady state in 1901, but after 1901 the pools and simulated flux drift in response to climate. The magnitude and range of simulated Hg^0^ flux and Hg^II^ export appear consistent with observed values^38^ (Supplementary Figs. 10, 11, and 12).

## Supplementary information

Supplementary Information

Supplementary Data

## Data Availability

This article includes all data generated or used during this study in its Supplementary Dataset files.
