# Supplementary material for: Potential impacts of mercury released from thawing permafrost
Source: Nat Commun. 2020 Sep 16;11:4650. doi: 10.1038/s41467-020-18398-5 (PMC7494925; doi:10.1038/s41467-020-18398-5)
Supplement: Supplementary file 1 — Supplementary Information [file 41467_2020_18398_MOESM1_ESM.pdf]

Supplementary Information: Potential impacts of mercury released from thawing permafrost

Kevin Schaefer[1], Yasin Elshorbany[2], Elchin Jafarov[3], Paul F. Schuster[4], Robert G. Striegl[4], Kimberly P. Wickland[4], Elsie M. Sunderland[5]

[1] National Snow and Ice Data Center, Cooperative Institute for Research in Environmental Sciences, University of Colorado Boulder, Boulder, CO, USA,

[2] College of Arts and Sciences, University of South Florida, St. Petersburg, Fl, USA

[3] Computational Earth Sciences, Los Alamos National Laboratory, Los Alamos, NM, USA,

[4] U.S. Geological Survey, Water Mission Area, Earth Systems Process Division, Boulder, CO, USA,

[5] Harvard John A. Paulson School of Engineering and Applied Sciences and Harvard T.H. Chan School of Public Health, Harvard University, Cambridge, MA, USA.

Correspondence and requests for materials should be addressed to K. S. (email: kevin.schaefer@nsidc.org)

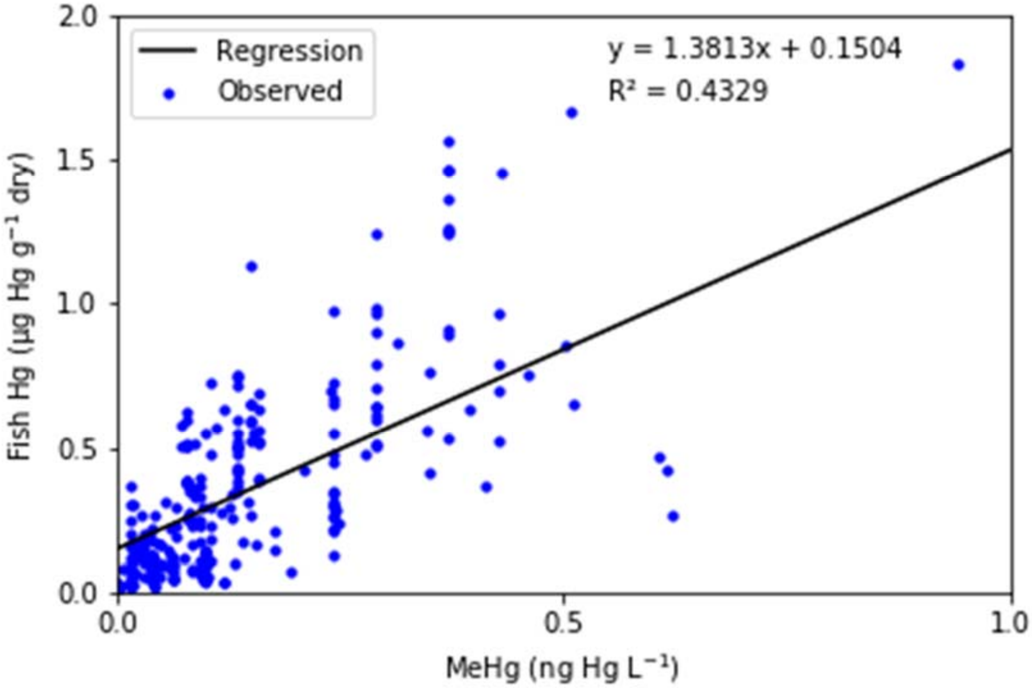

Supplementary Figure 1: Mercury (Hg) concentration in fish. The dry weight concentration of Hg in fish (Hg_{fish}) as a function of methyl mercury (MeHg) concentration in water. The plot shows the regression model used to estimate Hg_{fish} from our simulated MeHg concentrations shown in Supplementary Figure 11. We used 268 data points and the regression is statistically significant at the 95% level. We converted from dry weight to wet weight assuming fish mass is 80% water[1]. We include data from multiple fish species, so the regression model represents an average fish species[1, 2]. The regression model will differ for specific fish species.

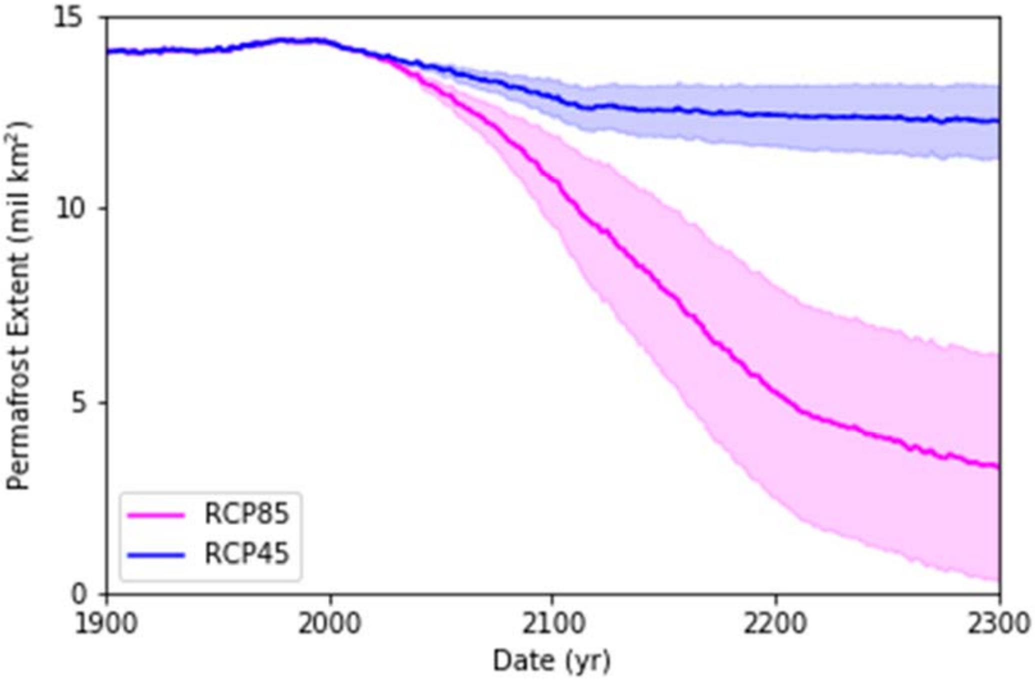

Supplementary Figure 2: Simulated decrease in permafrost extent. Permafrost extent is the area where simulated permafrost occurs in the top five meters of soil. RCP45 shows a modest decrease in near surface permafrost extent. RCP85 shows a near complete thaw of all near surface permafrost. The shaded area represents uncertainty in permafrost extent due to variability in projected climate.

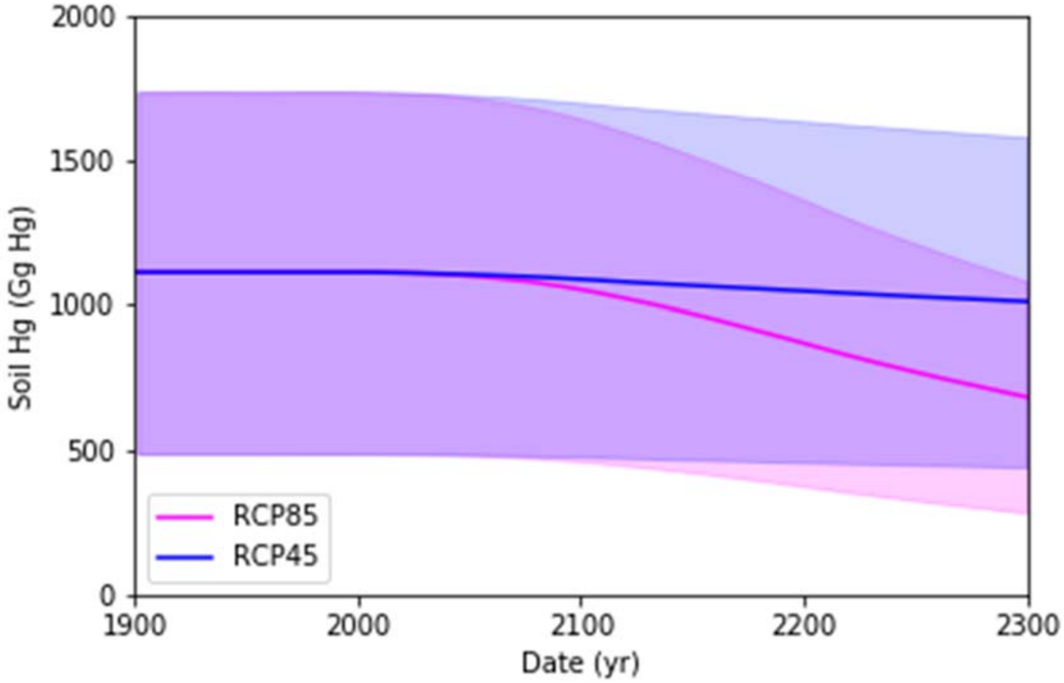

Supplementary Figure 3: Total soil mercury (Hg). Total soil Hg is the sum of thawed Hg in the active layer plus frozen Hg in permafrost. The shaded areas represent uncertainty. The total Hg stays relatively constant for most of the 20th century, consistent with our assumption of steady state initial conditions in 1901.

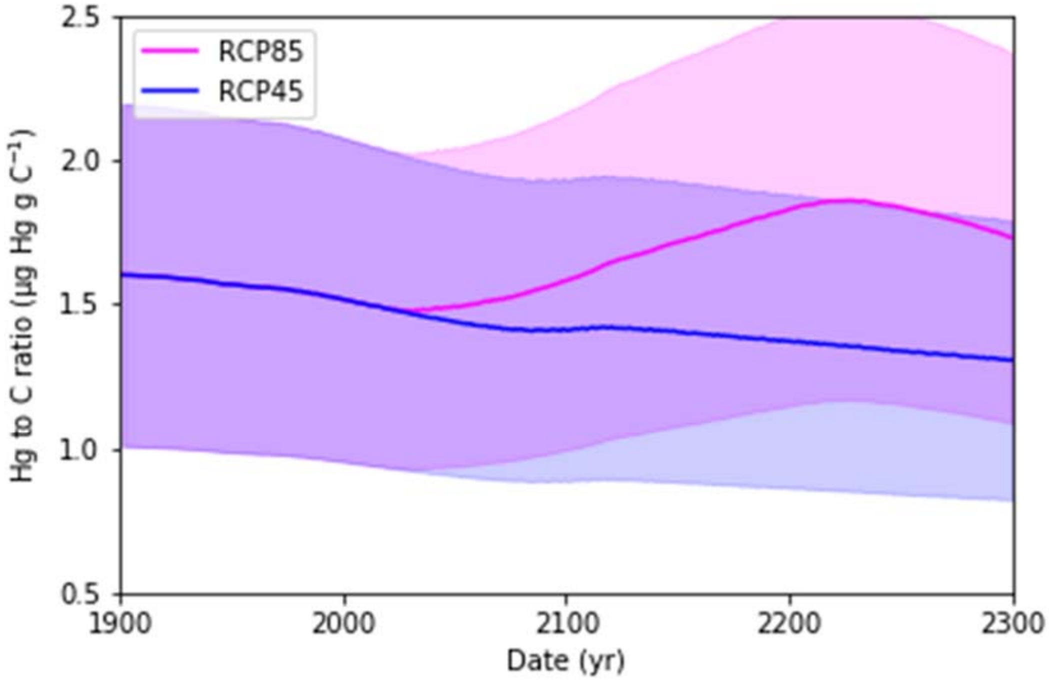

Supplementary Figure 4: The mercury to carbon ratio (Hg:C). The simulated Hg:C in the active layer starts at 1.6 µg Hg gC^{-1} consistent with using observations to initialize the model[3]. The shaded regions represent uncertainty. Hg:C for frozen organic matter in permafrost stays constant at 1.6 µg Hg gC^{-1}. Hg:C depends on the balance between carbon and Hg losses and gains. Hg losses are Hg^0 evasion and Hg^{II} leaching and gains are atmospheric deposition. Carbon decreases due to decay, but increases due to photosynthesis. The total amount of Hg within the soil column stays relatively constant over the 20th century, but Hg:C decreases because the total amount of biomass increases due to increased photosynthesis from higher temperatures and increasing atmospheric carbon dioxide. This decreasing trend continues for RCP45. For RCP85, however, Hg:C increases in the middle of the 21st century when carbon losses due to increased decay exceed enhanced photosynthesis.

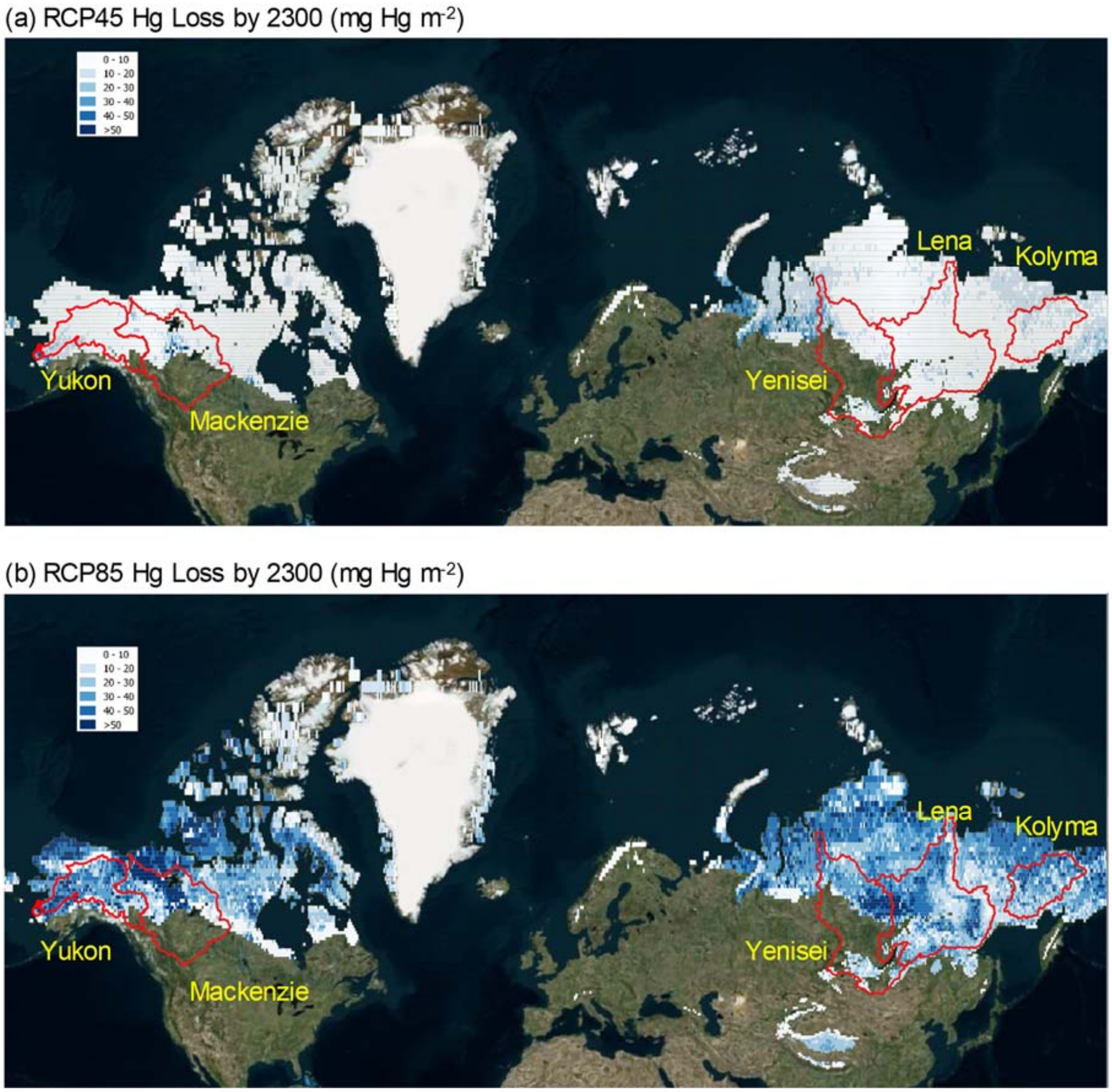

Supplementary Figure 5: Cumulative mercury (Hg) loss per area by 2300. Total Hg loss is net elemental mercury (Hg^0) flux to the atmosphere plus mercury cation (Hg^{II}) export by rivers summed from 1901 to 2300. The major river drainage basins appear outlined in red. Areas with the highest Hg losses represent permafrost regions most vulnerable to thaw with the largest amount of frozen carbon. All the major river basins show similar patterns of minimal releases for RCP45 and large releases for RCP85. The upper Mackenzie and Yenisei drainage basins lie outside the permafrost domain, which may modulate the effects of Hg release from permafrost.

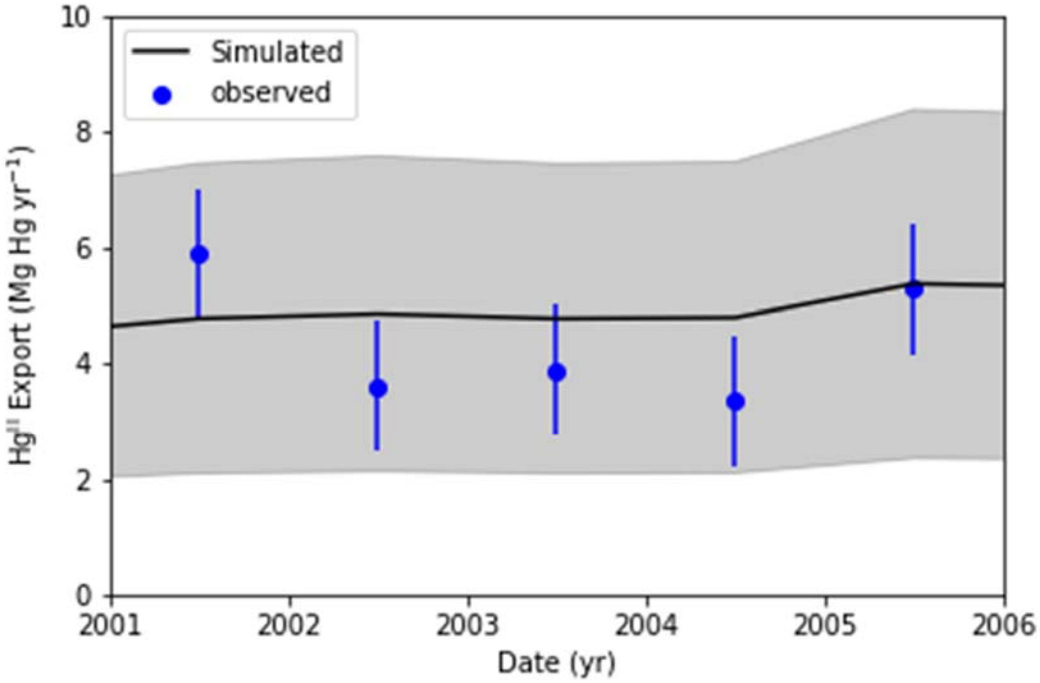

Supplementary Figure 6: Mercury cation (Hg^{II}) export from the Yukon River. Simulated annual Hg^{II} export from the Yukon River Basin (YRB) appears consistent in magnitude with observed values at Pilot Station, Alaska[4]. The shaded areas represent uncertainty in simulated values. The observed values represent annual averages with the standard deviation representing uncertainty. Pilot Station lies near the mouth of the Yukon River and the observed Hg^{II} export represents the integrated, total Hg^{II} riverine export for the entire YRB.

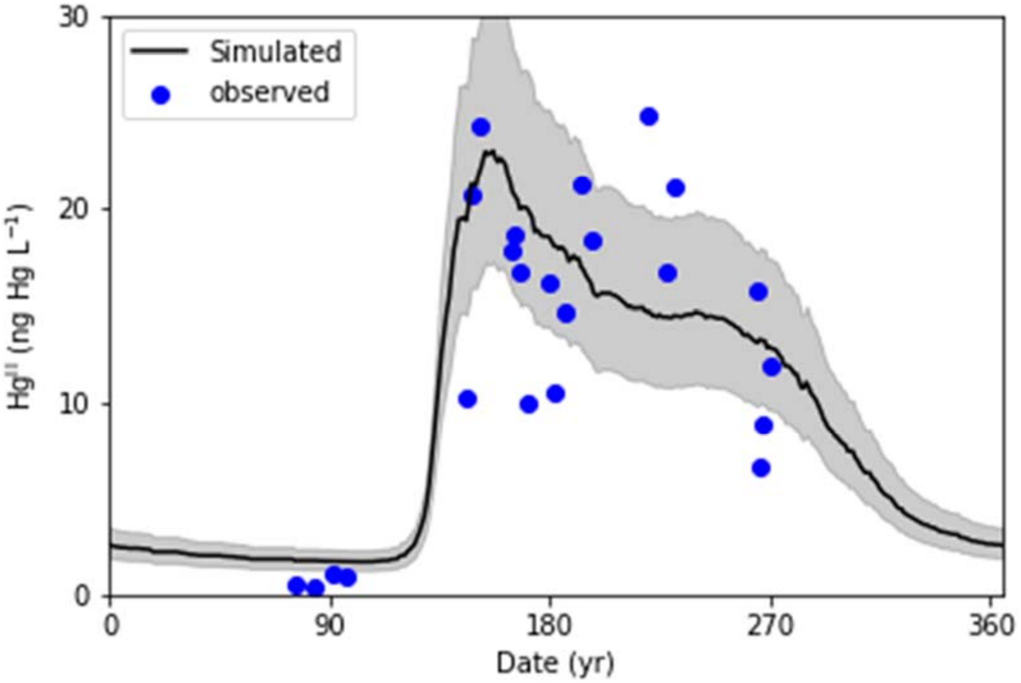

Supplementary Figure 7: Mercury cation (Hg^{II}) concentrations in the Yukon River. Simulated Hg^{II} concentrations (ng Hg L^{-1}) appear consistent with observed values at Pilot Station, Alaska, near the mouth of the Yukon River[4]. The observations span the time period of 2001 through 2005 and the simulated concentrations represent the average seasonal cycle from 2001 through 2005. The shaded areas represent uncertainty in the simulated concentrations.

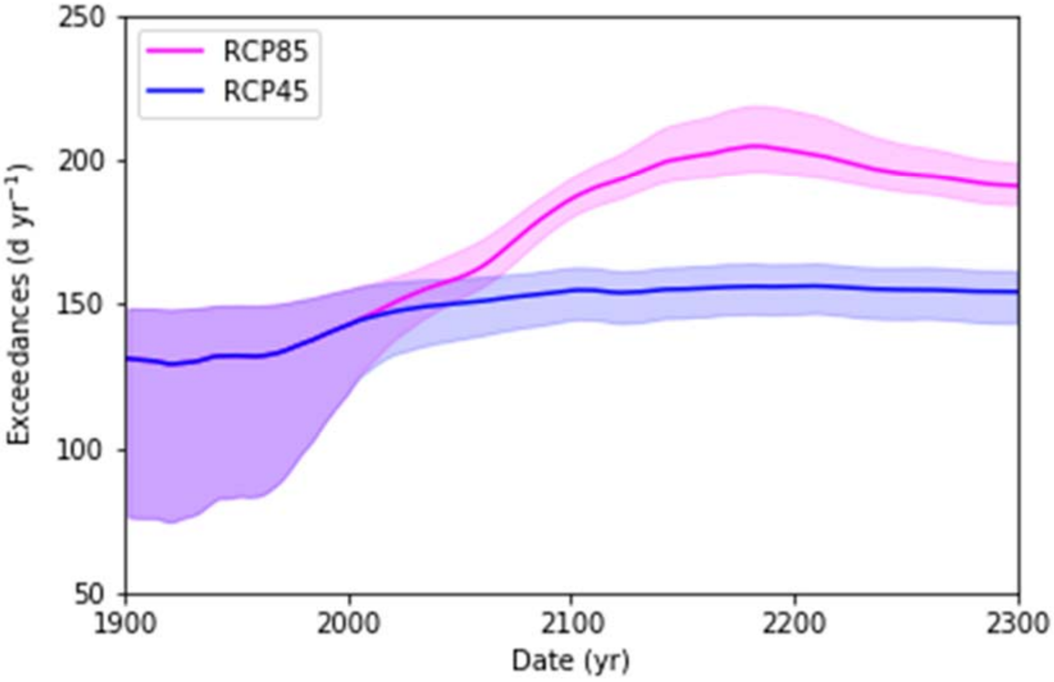

Supplementary Figure 8: Water quality exceedances. Exceedance is the number of days per year that estimated mercury cation (Hg^{II}) concentration at Pilot Station exceeds the Environmental Protection Agency (EPA) water quality criterion of 12 ng Hg L^{-1}. The solid lines represent the means and the shaded areas represent uncertainty. We applied a 20-year running mean filter to reduce noise.

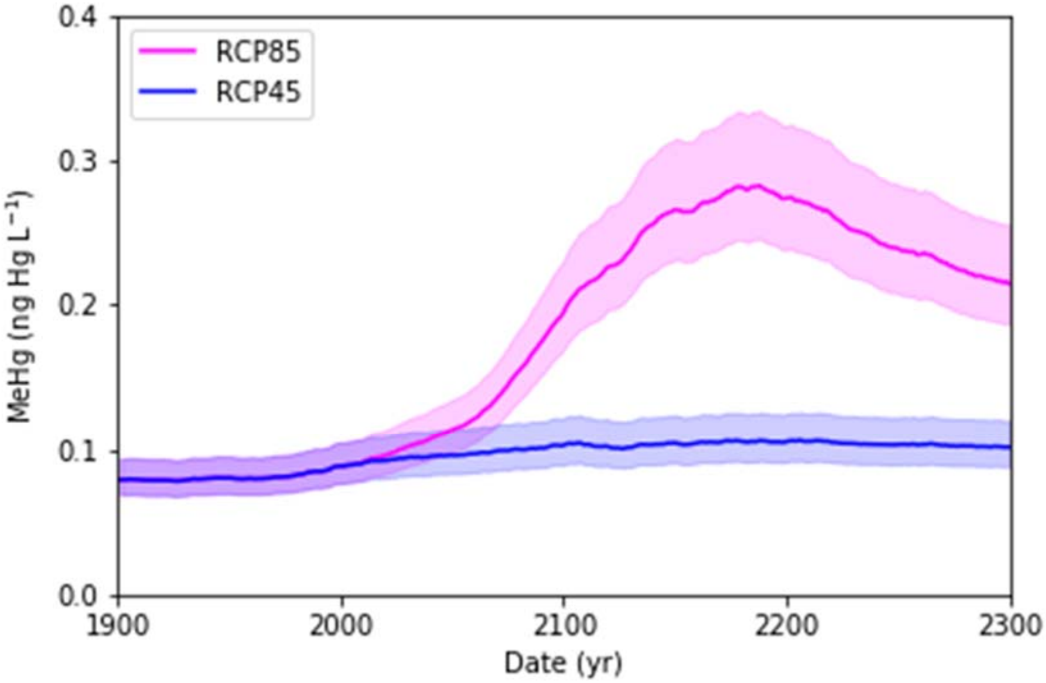

Supplementary Figure 9: Methyl mercury (MeHg) concentrations. Annual average, simulated MeHg concentrations in the Yukon River at Pilot Station, Alaska. The shaded areas represent uncertainty. We first calculated annual average mercury cation (Hg^{II}) concentrations and applied a 10-year running mean filter to reflect the fact that fish accumulate MeHg over their lifetimes and the concentration does not go up and down with seasonal and interannual variability. We then multiplied the smoothed annual average Hg^{II} concentration by the observed MeHg to Hg^{II} ratio of 1%[4].

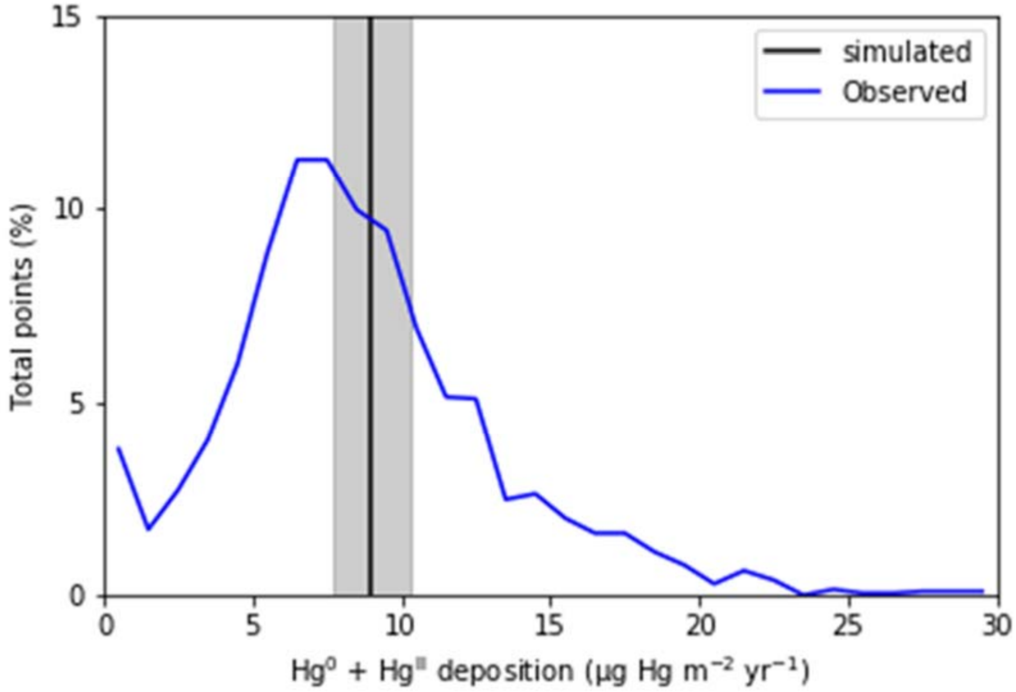

Supplementary Figure 10: Probability distribution of mercury (Hg) deposition. Steady state initial conditions in 1900 required a total, pan-Arctic Hg deposition rate of 0.75±0.11 Gg Hg yr^{-1} or 9.0±1.3 µg Hg m^{-2} yr^{-1}, shown here as the vertical line with the shaded area representing uncertainty. We compared to Hg^{II} wet deposition data from the Mercury Deposition Network (MDN) (http://nadp.slh.wisc.edu/MDN/) and dry Hg^0 deposition on the North Slope of Alaska[5]. The MDN included 39 measurements of wet deposition for Alaska collected over several years with an average and standard deviation of 2.6±1.7 µg Hg m^{-2} yr^{-1}. SiBCASA assumes 71% Hg^0 dry deposition and 29% Hg^{II} wet deposition based on observations[9], resulting in a simulated annual dry Hg^0 deposition of 6.4±0.9 µg Hg m^{-2} yr^{-1} and an annual wet Hg^{II} deposition of 2.6±0.4 µg Hg m^{-2} yr^{-1}, consistent with the observed value from the MDN. Here, we compare the simulated Hg deposition to a PDF of all 2071 observed deposition rates in the MDN. The simulated total deposition rate of 9.0±1.3µg Hg m^{-2} yr^{-1} appears consistent with the mean deposition of 8.6±45 µg Hg m^{-2} yr^{-1} based on observed deposition rates across North America.

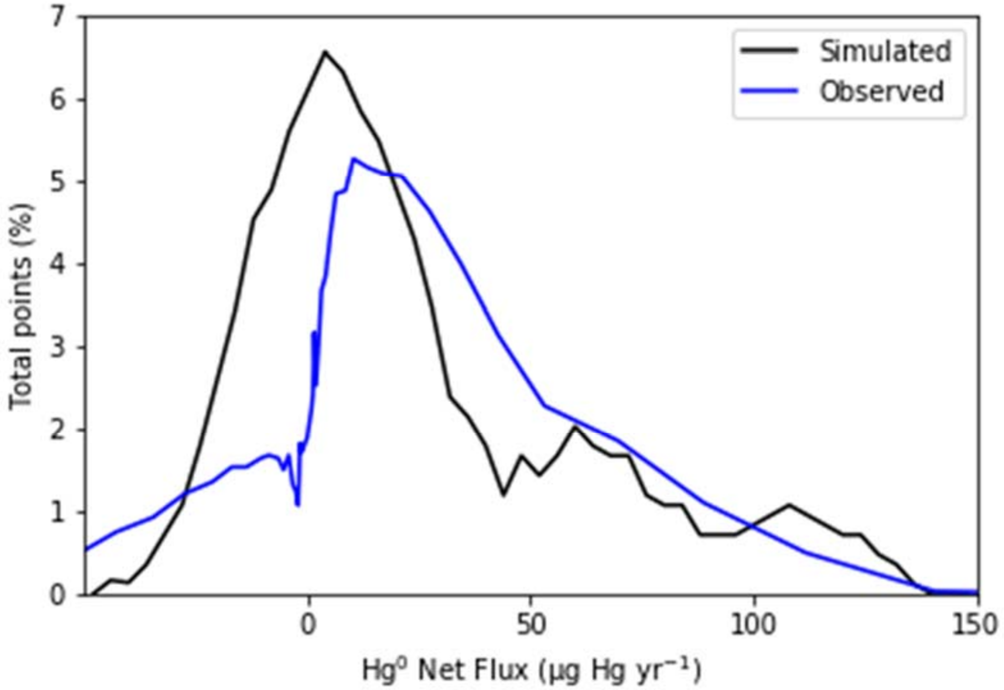

Supplementary Figure 11: Distribution of modern net elemental mercury (Hg^0) flux. Simulated net Hg^0 fluxes for the Yukon River Basin (YRB) between 1900 and 2020 appear consistent in magnitude and range with observed Hg^0 fluxes worldwide. The net flux is evasion minus deposition, with a positive value indicating a net release into the atmosphere. A compilation of 132 studies included nearly all published measurements of net Hg^0 flux, but only 4 of the studies focused on permafrost[6]. To provide a more comprehensive model evaluation, we compared a Probability Density Function (PDF) of simulated net Hg^0 fluxes in the YRB to a PDF of all available observations from background, undisturbed sites. Both have peaks or modes at slightly positive values of net Hg^0 flux, indicative of net Hg release to the atmosphere. The model started at steady state in 1900, but transitions to an Hg source by the 1940s, resulting a peak or mode at an Hg^0 flux of 6 μg Hg m^{-2} yr^{-1}. Both show skewed distributions towards positive fluxes.

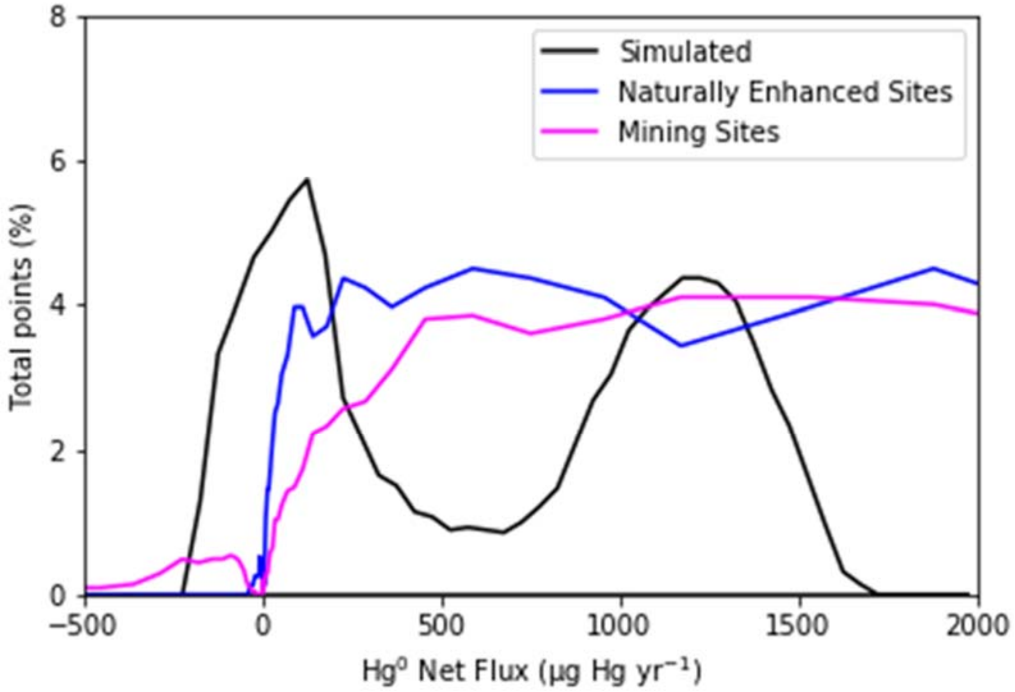

Supplementary Figure 12: Distribution of future net elemental mercury (Hg^0) flux. Simulated net Hg^0 fluxes for the YRB between 1900 and 2300 for the RCP 8.5 scenario appear consistent with observed Hg^0 fluxes for contaminated sites [Agnon et al., 2016]. Here, we compare a Probability Density Function (PDF) of simulated net Hg^0 fluxes between 1900 and 2300 for the YRB with PDFs of observed fluxes for naturally enhanced fluxes from enriched geologic sites and anthropogenically enhanced fluxes from contaminated mining sites[6]. Both naturally enriched and contaminated mining sites show observed Hg^0 flux values often exceeding 10,000 µg Hg m^{-2} yr^{-1}. The simulated fluxes show two peaks, a primary peak at ~100 µg Hg m^{-2} yr^{-1} a secondary peak at ~1200 µg Hg m^{-2} yr^{-1}. The primary peak results from simulated fluxes early in the simulation when the YRB was still near steady state with relatively low fluxes. The secondary peak results from simulated fluxes after 2100 when the permafrost thaws out and the organic matter decays, consistent with observed fluxes from contaminated sites. Although thawed, the organic matter decays slowly with a characteristic time scale of 75 years, limiting evasion rate such that the simulated Hg^0 flux rarely exceeds ~1600 µg Hg m^{-2} yr^{-1}.

Supplementary References

1. Scudder, E., Riva-Murray, J., Knightes, C. D., Journey, C. A., Chasar, L. C., Brigham, M. E., Bradley, P. M. Optimizing fish sampling for fish-mercury bioaccumulation factors. Chemosphere 135, 467-473, http://dx.doi.org/10.1016/j.chemosphere.2014.12.068 (2015).

2. Brumbaugh, W. G., Krabbenhoft, D. P., Helsel, D. R., Wiener, J. G., & Echols, K. R. A National Pilot Study of Mercury Contamination of Aquatic Ecosystems along Multiple Gradients: Bioaccumulation in Fish, Biological Science Report. USGS/BRD/BSR-2001-0009 (2001).

3. Schuster, P. F., Schaefer, K. M., Aiken, G. R., Antweiler, R. C., Dewild, J. F., Gryziec, J. D., Gusmeroli, A., Hugelius, G., Jafarov, E., Krabbenhoft, D. P., Liu, L., Herman-Mercer, N., Mu, C., Roth, D. A., Schaefer, T., Striegl, R. G., Wickland, K. P., & Zhang, T. Permafrost stores a globally significant amount of mercury. Geophys. Res. Lett., 45, 1463-1471. https://doi.org/10.1002/2017GL075571 (2018).

4. Schuster, P. F., Striegl, R. G., Aiken, G. R., Krabbenhoft, D. P., Dewild, J. F., Butler, K., Kamark, B., & Dornblaser, M. Mercury Export from the Yukon River Basin and Potential Response to a Changing Climate. Environ. Sci. Technol., 45, 9262-9267, dx.doi.org/10.1021/es202068b (2011).

5. Obrist, D., Agnan, Y., Jiskra, M., Olson, C. L., Colegrove, D. P., Hueber, J., Moore, C. W., Sonke, J. E., & Helmig, D. Tundra uptake of atmospheric elemental mercury drives Arctic mercury pollution. Nature, 547, 201-204, doi:10.1038/nature22997 (2017).

6. Agnan, Y., Le Dantec, T., Moore, C. W., Edwards, G. C., & Obrist, D. New Constraints on Terrestrial Surface Atmosphere Fluxes of Gaseous Elemental Mercury Using a Global Database. Environ. Sci. Technol., 50, 507-524, DOI: 10.1021/acs.est.5b04013 (2016).
